# Effect of Phosphate-Bridged Monomer on Thermal Oxidative Behavior of Phthalonitrile Thermosets

**DOI:** 10.3390/polym16162239

**Published:** 2024-08-07

**Authors:** Marina Sergeevna Lobanova, Alexandr Vladimirovich Babkin, Alexey Valeryevich Kepman, Victor Vasil’evich Avdeev, Oleg Sergeevich Morozov, Boris Anatol’evich Bulgakov

**Affiliations:** Department of Chemistry, M. V. Lomonosov Moscow State University, 119991 Moscow, Russia

**Keywords:** phthalonitrile resins, thermosets, thermal oxidative aging, mechanical properties, thermal oxidative stability

## Abstract

Phthalonitrile thermosets are known for their excellent mechanical, physico-chemical, and fire-retardant properties, making them attractive for aerospace and mechanical engineering applications. When producing and applying phthalonitrile-based structural parts, it is essential to consider aspects such as processability and the long-term stability of the material’s properties at high temperatures. In our previous studies, we demonstrated that resins containing phosphate-bridged bisphthalonitrile monomers are easily processable due to their low melting temperature and wide processing window. In this study, we investigated the impact of bis(3-(3,4-dicyanophenoxy)phenyl)phenyl phosphate (PPhPN) monomer content on physico-chemical and mechanical properties, thermal stability, and thermal oxidative stability. This research highlights the importance of conducting long-term thermal oxidative aging studies in addition to thermogravimetric analysis to properly assess the stability of thermosets. The findings indicate that adding less than 15% of PPhPN results in the formation of a crystalline phase, which impairs the resin’s processability. Conversely, a high PPhPN content reduces the material’s thermal oxidative stability. Therefore, based on mechanical and physico-chemical tests after thermal oxidative aging, it can be concluded that a 10–15% concentration of the phosphate-containing monomer enables easy processability of the phthalonitrile resin and provides excellent long-term thermal oxidative stability at temperatures up to 300 °C, while maintaining a flexural strength exceeding 120 MPa and an elasticity modulus of 4.3 GPa.

## 1. Introduction

Phthalonitrile-based materials have attracted significant attention from the aerospace and mechanical engineering industries due to their exceptional heat resistance properties [1]. These materials are characterized by high glass transition temperatures exceeding 400 °C [2,3,4], decomposition temperatures (T5%) over 500 °C [5,6], outstanding mechanical properties [7,8], low water absorption below 3% [6,9], and Limiting Oxygen Index (LOI) values of up to 50% [10,11], which contribute to their remarkable fire-retardant characteristics [10,12,13,14] and expand their potential applications as structural materials. The development of these properties is attributed to the formation of a three-dimensional cross-linked polymer network comprising triazine, phthalocyanine, and isoindoline structures during the curing process [15,16,17,18].

The thermal oxidative stability of polymers is a crucial challenge to address in operations conducted at elevated temperatures. Short-term experiments have demonstrated that phthalonitrile composites have shown the ability to retain up to 60% of their mechanical properties at temperatures up to 400 °C [19,20,21,22]. However, phthalonitrile matrices are prone to oxidation at such elevated temperatures [13,23,24]. Enhancing the thermal oxidative stability of phthalonitrile resins can be achieved through the optimization of curing and post-curing processes [4], careful selection of curing agents [25,26], and modification of the chemical structure of phthalonitrile monomers [27,28,29]. The incorporation of organoelement linkers into the monomer backbone has been identified as a strategy to enhance oxidative stability at elevated temperatures [30,31]. Additionally, modifiers such as phosphorus [30,32], fluorine [16,33], and silicon [14,29,32] can be utilized to enhance the properties of phthalonitrile monomers.

The utilization of silicon is based on the principle that oxygen and free radicals break the carbon–silicon bond, leading to the formation of siloxy moieties. These moieties produce an inert phase and reduce the thermal oxidation process [31,34]. In a study [35], the impact of fluorine on the thermal stability of phthalonitrile resin is discussed. The authors hypothesized that fluorinated resins would exhibit higher resistance to oxidation due to better oxidation resistance than the C—H bond. The thermogravimetric analysis (TGA) results indicated slightly better stability of the fluorinated resin, with a 5% mass loss temperature (T5%) of 443.7 °C compared to the T5% of 438.5 °C observed for novolac-phthalonitrile resin. Additionally, findings from a study [16] on fluorinated curing agents also demonstrated an increase in the stability of the resins, with the determined T5% reaching up to 520 °C.

Phosphorus-containing compounds among other modifiers may be more attractive due to the combination of properties such as high flame-retardancy [36,37,38,39,40,41] and enhanced technological characteristics. Previous studies [4,10,19,20,32,42] indicated that the addition of phosphate-containing monomers enhances the processability of phthalonitrile resins by reducing melting points and complex viscosities of the resin melts, enabling the utilization of cost-effective techniques for manufacturing phthalonitrile composites. Additionally, based on the TGA data, the use of phosphate-based linkers reduces the thermal oxidation rate [32].

Selecting an appropriate method to enhance thermal oxidative stability is crucial, as is the selection of a suitable evaluation technique. Currently, thermogravimetric analysis (TGA) is a commonly utilized method for determining mass loss within a specific temperature range. However, as demonstrated in our previous studies [43,44], these parameters are only suitable for rapid initial comparison of materials and do not accurately reflect the long-term thermal oxidative stability of resins. Therefore, this study proposes the utilization of long-term thermal-oxidative aging to evaluate the operational limitations of the materials.

This article investigates the influence of the content of a phosphate-bridged bisphthalonitrile monomer, bis(3-(3,4-dicyanophenoxy)phenyl)phenyl phosphate [25], on the physico-chemical and mechanical characteristics of phthalonitrile resins, along with their thermal oxidation resistance. This analysis enables the anticipation of operational limitations of phthalonitrile thermosets and polymer composites derived from them.

## 2. Materials and Methods

### 2.1. Materials

Resorcinol, 4-nitrophtalonitrile, 4-cyanophenol, potassium carbonate (Acros Organics, Geel, Belgium) were used as received. Solvents dimethylacetamide (DMAA), dichloromethane (DCM), toluene, methanol were purchased from Alfa Aesar Company (Haverhill, MA, USA). The phthalonitrile monomers 1,3-bis(3,4-dicyanophenoxy)benzene (DPB) [45], bis(3-(3,4-dicyanophenoxy)phenyl)phenyl phosphate (PPhPN) [32], and 4-aminophenoxyphthalonitrile (APN) [46], were synthesized according to the published procedures.

### 2.2. Sample Preparation

**Figure 1:**
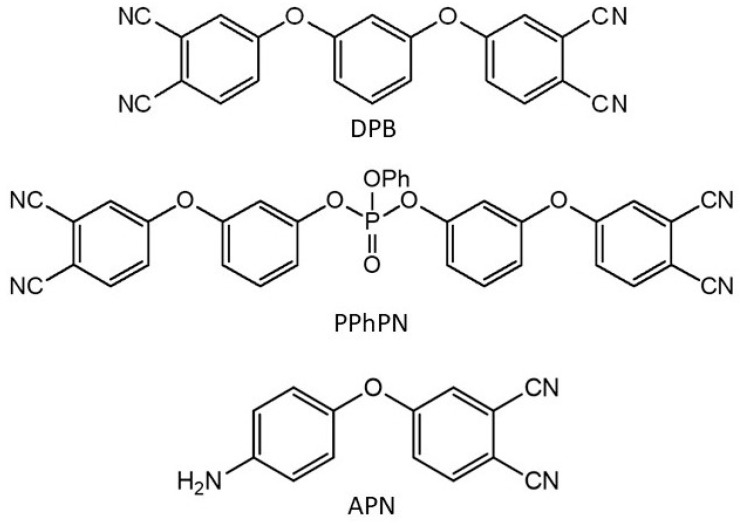
Structures of monomers.

For phthalonitrile resins preparation, we used a commonly known phthalonitrile monomer, 1,3-bis(3,4-dicyanophenoxy)benzene (DPB), a reactive diluent for phthalonitrile monomers, bis(3-(3,4-dicyanophenoxy)phenyl)phenyl phosphate (PPhPN), and a curing agent, 4-aminophenoxyphthalonitrile (APN). Structures of monomers used in this research are shown in Figure 1.

The resin components DPB and PPhPN in the ratios shown in Table 1 were mixed in a 2L reactor equipped with a mechanical stirrer. The mixture was subjected to a vacuum at 180 °C until it melted and underwent complete degassing. Subsequently, the temperature was reduced to 160 °C, and the third component, APN, was added in the proportions detailed in Table 1. The mixture was then stirred and degassed thoroughly. The total mass of the resulting resin was 400 g.

The liquid resin was prepared and placed in a metal mold measuring 320 × 500 × 2 mm, then cured at 180 °C for 8 h. Subsequently, the cured samples were post-cured free-standing in a fine char powder at 350 °C for 8 h. The phthalonitrile resin, which contained varying percentages of PPhPN (50%—PN50, 30%—PN30, 15%—PN15, 10%—PN10), was labeled accordingly. For thermogravimetric analysis (TGA) tests, the post-cured resin samples were ground in a mortar. Samples for dynamic mechanical analysis tests (50 × 5 × 2 mm) and flexural strength tests (50 × 10 × 2 mm) were cut using computer numerical control (CNC) equipment.

### 2.3. Methods

Glass transition temperatures were determined by dynamic mechanical analysis (DMA) using a DMA Q800 instrument (New Castle, DE, USA) under nitrogen atmosphere within the temperature range of 200–500 °C at a frequency of 1 Hz.

Flexural properties were determined by 3-point bending method with a 50ST testing machine (Tinius Olsen Ltd., Salfords, UK) in accordance with ASTM D790 [47].

TGA analysis was conducted using an STA 449 F5-Jupiter thermogravimetric analyzer (NETZSCH-Gerätebau GmbH, Selb, Germany) with powder resin samples (10.0 ± 0.1 mg) placed in alumina crucibles. The analysis was performed in the temperature range of 50–1000 °C with the heating rate of 10 °C/min under air atmosphere with the gas flow rate of 60 mL/min. The processing of experimental curves was carried in Proteus Analysis 7.1 software.

To estimate the long-term thermal oxidative stability of resin samples for DMA and flexural testing, the samples were subjected to thermal aging at temperatures of 300 °C, 330 °C, and 350 °C for the duration of 200 h. An examination of parameters such as mass loss, glass transition temperature (Tg), flexural strength, and crack propagation was conducted.

The mass loss resulting from thermal aging was determined by measuring the weight using OHAUS AP 250D scales (Union, NJ, USA).

Scanning electron microscopy (SEM) images were taken on a Tescan Vega 3 electron microscope (Tescan, Brno, Czech Republic) at 20 kV.

## 3. Results and Discussion

### 3.1. Resin Synthesis and Characterization

To estimate the impact of components on resin properties, samples containing 10%, 15%, 30%, and 50% of PPhPN were prepared.

In order to facilitate cost-effective injection methods, the resins must possess an amorphous structure. Initially, the blends were analyzed using the differential scanning calorimetry (DSC) technique. The data revealed that the presence of a crystalline phase was observed at 10% PPhPN content, as evidenced by an endothermal peak at 158 °C (Figure S1). Conversely, DSC curves of blends containing 15–50% PPhPN showed no additional peaks indicating good compatibility of the components and an amorphous state of the resins (Figure S2).

The resins with different concentrations of PPhPN were post-cured at 350 °C. Subsequently, the resulting thermosets were studied using DMA, TGA, and flexural tests to evaluate the impact of the component on the thermosets’ properties (Table 2).

The analysis of the DMA data indicates that an increase in PPhPN content led to the formation of stiffer structure, as evidenced by a 20 °C increase in the glass transition temperature from PN10 to PN50 (Figure S3). The results of the flexural tests showed no significant difference in strength among the examined samples. However, the flexural moduli increased with PPhPN content, thereby validating the formation of more rigid polymer networks. The flexural moduli (Ef) for PN10 and PN15 were 4.3 ± 0.1 GPa, while for PN30 it was 4.4 ± 0.1 GPa, and for PN50 it was 4.7 ± 0.1 GPa, indicating the formation of stiffer polymers.

**Figure 2:**
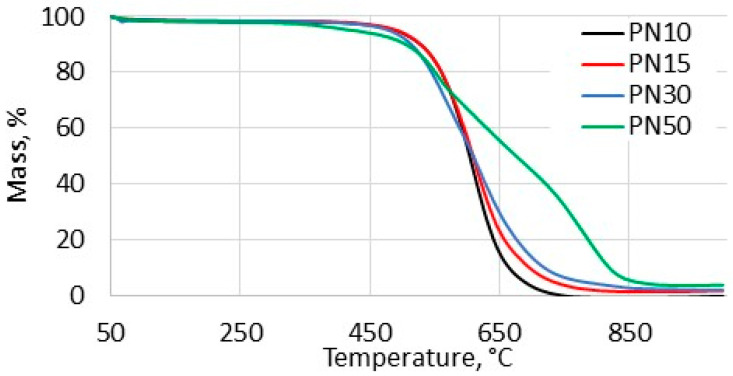
TGA curves of the phthalonitrile resins under air atmosphere.

The results of the TGA (Table 2, Figure 2) indicated that the phthalonitrile resins under investigation exhibited higher susceptibility to thermal oxidation with increased PPhPN content. The temperature corresponded to 5% mass loss under air atmosphere (TOS5%) decreased sequentially for PN10, PN15, PN30, PN50, with respective temperatures of 507 °C, 507 °C, 498 °C, and 470 °C, indicating the formation of less thermally stable networks as the PPhPN concentration in the resin increased. It is noteworthy that powdered samples were used in the TGA experiments to mitigate surface effects on oxidation. All samples underwent complete combustion by the end of the experiments, in contrast to prior findings that demonstrated incomplete combustion of phosphate-containing thermosets during TGA tests [32]. However, the oxidation rate in the later stages (above 500 °C) decreased with an increase in the phosphorus-containing component concentration (Figure 2).

According to previous studies, the TOS5% value should be viewed as a preliminary estimate of thermal oxidative stability [43]. Therefore, additional long-term isothermal tests were conducted to further investigate the impact of PPhPN on the thermal oxidation resistance of the thermosets.

### 3.2. Thermal Aging of the Thermosets

**Figure 3:**
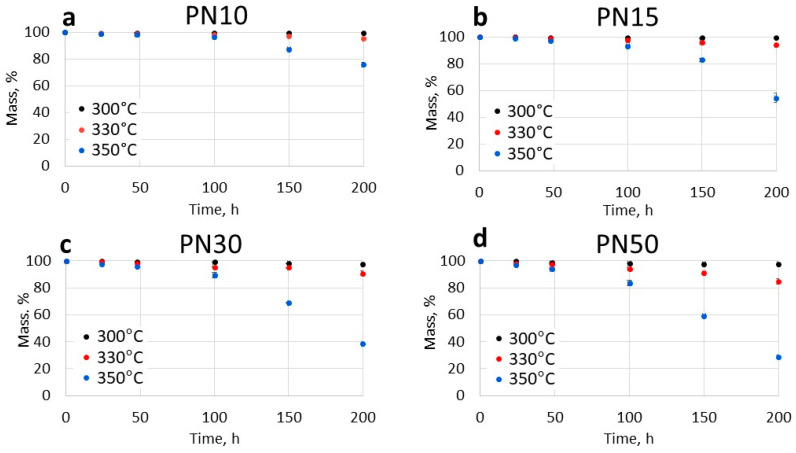
Mass loss curves of the phthalonitrile resins during thermal oxidative aging: (**a**)—PN10 resin, (**b**)—PN15 resin, (**c**)—PN30 resin, (**d**)—PN50 resin.

Thermal oxidative aging experiments were carried out at temperatures of 300 °C, 330 °C, and 350 °C for 200 h to evaluate the operational parameters and service life of phthalonitrile materials under high-temperature conditions. Specimens of standard dimensions (50 × 10 × 2 mm) were used in this study. The mass loss results are presented in Figure 3.

Based on the mass loss data (Figure 3), it can be concluded that all of the studied resins are characterized by low degradation rate at 300 °C. Specifically, PN10 and PN15 showed a mass loss of less than 1%, while PN30 and PN50 lost about 3% after 200 h of thermal oxidative aging. As the aging temperature increased, the influence of PPhPN content in the resin became more prominent. For instance, following 200 h of aging at 330 °C, samples containing 10% and 15% of PPhPN lost 5% and 6% of their mass, respectively, whereas PN30 and PN50 lost 9% and 15%, respectively (Table 2, Figure 2). Higher susceptibility to thermal oxidation observed in resins with higher PPhPN content suggests that the degradation of the polymer structure predominantly occurs in the phosphate groups [48]. It is probable that aryl phosphates decompose thermally into oligomeric polyphosphates, non-volatile polyphosphoric acid, and volatile organic compounds (H2O, phenol) through an elimination step, leading to the formation of reactive monomers and arynes (Figure S4) [49]. Previous research [43] has identified the production of phenol and aniline during the thermal aging of PN resin containing PPhPN under vacuum conditions using NMR and LC/MS. This degradation mechanism can be extrapolated to the PN10–50 resins. The recent research have shown the presence of the polyphosphate structure even after carbonization of phosphate-containing phthalonitrile resins at 1000 °C [50].

The results of DMA tests carried out post-aging (Table 3) revealed a rise in glass transition temperatures, indicating further structural changes in the thermosets until destruction. This corresponds to a critical mass loss of approximately 15%, likely stemming from additional cross-linking reactions and rearrangement of the matrix after the elimination of non-carbon atoms [31], which accompanies degradation. After 200 h of aging at 300 °C, the glass transition temperature (Tg) increased by approximately 20 °C for all specimens. Subsequent to aging at 330 °C and 350 °C, the glass transition temperatures exceeded 400 °C. This phenomenon was also noted in our previous studies [43]. Conversely, the thermal degradation of epoxies led to a decrease in the glass transition temperature [51]. This confirms the simultaneous processes of polymer structural modification, leading to an escalation in crosslinking degree and thermal oxidative degradation, which can also be linked to the formation of polyphosphate structures.

**Figure 4:**
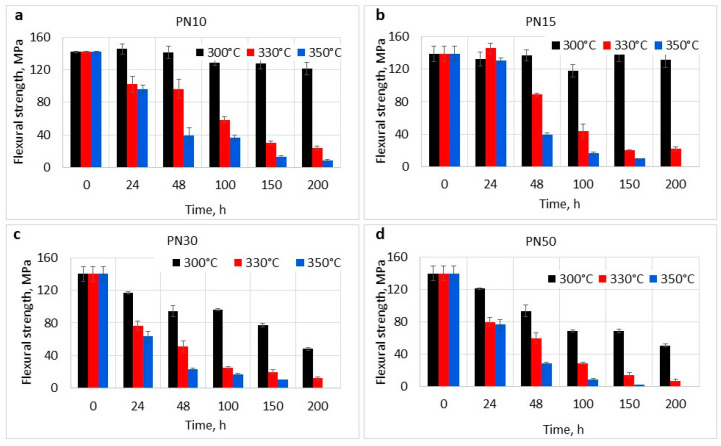
Flexural strength of phthalonitrile resins after oxidative aging: (**a**)—PN10 resin, (**b**)—PN15 resin, (**c**)—PN30 resin, (**d**)—PN50 resin.

**Figure 5:**
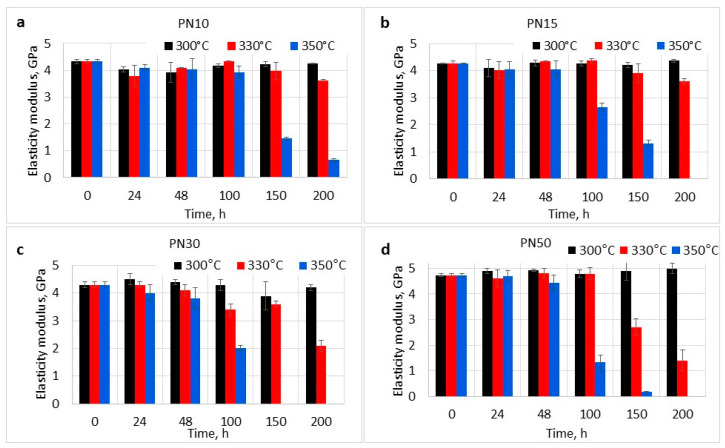
Elasticity moduli of phthalonitrile resins after oxidative aging: (**a**)—PN10 resin, (**b**)—PN15 resin, (**c**)—PN30 resin, (**d**)—PN50 resin.

The mass loss and the increase in glass transition temperature (Tg) signify alterations in the polymer’s structure. It is important to estimate the impact of these changes on the mechanical properties of the materials after aging. For this purpose, flexural tests were carried out on the aged samples (Figure 4 and Figure 5).

The results of mechanical tests showed that the flexural strength of the resins with low content of PPhPN retained over 85% of the initial value after 200 h of aging at 300 °C while the strength of PN30 and PN50 decreased by above 70%. After 200 h of aging at 300 °C, the values were 121.6 ± 7.6 MPa, 131.4 ± 4.4 MPa, 48.0 ± 1.8 MPa, 54.3 ± 6.3 MPa, respectively. At 330 °C and 350 °C, mechanical characteristics decreased rapidly, leading to an overall loss of flexural strength over 85% for all of the studied materials with the resulting values at 330 °C for PN10 of 24.1 ± 2.2 MPa, PN15—22.1 ± 2.7 MPa, and 11.9 ± 0.2 MPa, 7.3 ± 1.9 MPa for PN30 and PN50, correspondingly. At 350 °C, flexural strength of the thermosets drastically decreased, indicating that the materials may only be suitable for a short-term operation. Thus, polymers with a high content of PPhPN lost mechanical strength faster. This led us to conclusion that P−O−C bonds were the weakest link in a chain scission process [52]. Phosphate structures presumably formed polyphosphate structures during the phthalonitrile degradation process, which became more visible with an increase in PPhPN concentration [48].

The elasticity moduli (Figure 5) of the resins increased with higher PPhPN content, as previously mentioned. During the aging at 300 °C, the resins maintained high moduli values, indicating the retention of the materials stiffness. The moduli of the resins aged at 300 °C were 4.3 ± 0.1 GPa, 4.4 ± 0.1 GPa, 4.2 ± 0.1 GPa, and 5.0 ± 0.2 GPa, respectively, which was in accordance with the T_g_ growth during aging. During the aging at 330 °C and 350 °C, the decrease in elasticity moduli was observed for all of the studied materials after 10–15% mass loss, indicating the accumulation of critical number of defects. After 200 h of aging at 330 °C, the E_f_ of PN10 and PN15 decreased by 16% (3.6 ± 0.1 GPa) and 15% (3.6 ± 0.1 GPa), while the E_f_ of PN30 and PN50 decreased by more than 50%.

**Figure 6:**
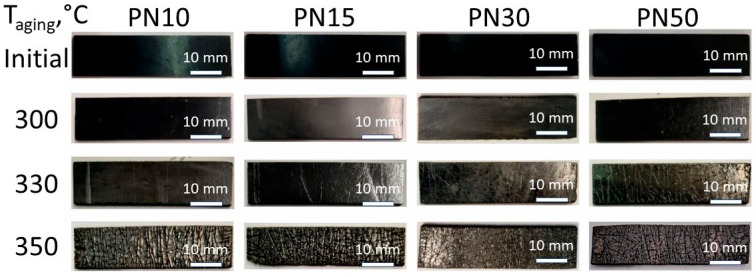
Images of resins PN10, PN15, PN30, and PN50: before and after 200 h of thermal oxidative aging at 300 °C, 330 °C, and 350 °C.

Visual inspection of the samples for flexural tests (Figure 6) confirmed the absence of significant defects both before and after aging at 300 °C for all the samples. At higher aging temperatures, cracks formed earlier in samples with higher PPhPN content. Aging at 330 °C led to the formation of surface cracks in PN30 and PN50 after 100 h, and in PN10 and PN15 after 150 h. Cracks started to appear after 48 h of aging at 350 °C, and after 100 h in PN15.

**Figure 7:**
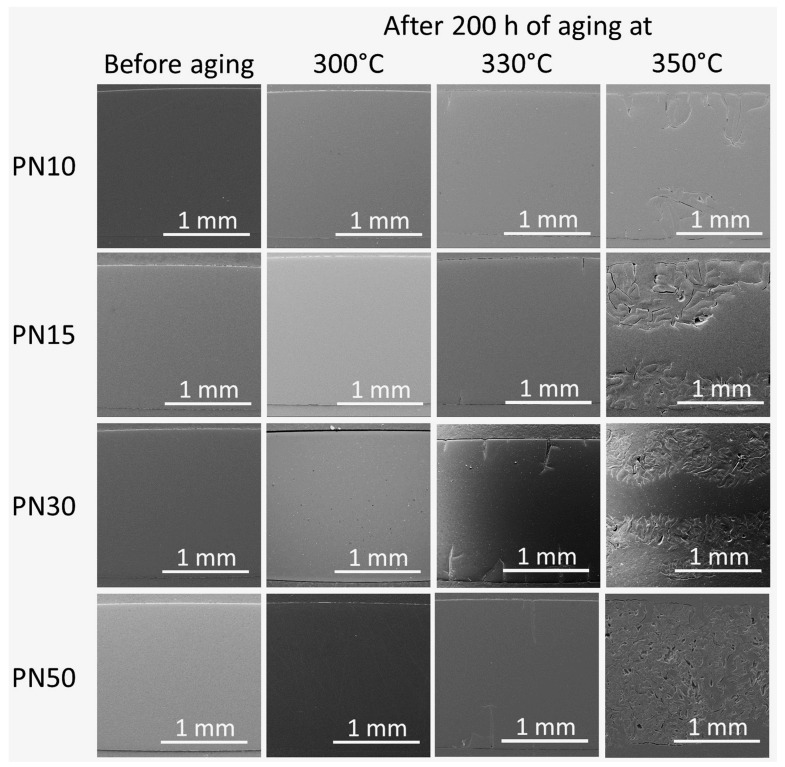
SEM micrographs of cross-section of the phthalonitrile resins before aging and after 200 h of aging at 300 °C, 330 °C, and 350 °C.

SEM study of the phthalonitrile resins (Figure 7) confirmed the growth of cracks not only on the surface of the resins but also within the depth of the sample. The sample showed no surface or volume defects before aging. After 200 h of aging at 300 °C, no defects were observed inside the samples. Aging at 330 °C for the same duration resulted in the expansion of cracks ranging from 200 to 250 µm in PN10 and PN15 and from 500 to 600 µm in PN30 and PN50. However, the number of cracks visible in the cross-section was not sufficient, indicating that the destruction primarily affected the surface and near-surface layers due to thermal oxidation rather than pyrolysis. After 200 h of aging at 350 °C, PN10 had significant number of cracks with lengths ranging from 700 to 800 µm, PN15 and PN30 had a thermo-oxidative layer with a depth of 850 µm and PN50 was completely destroyed.

## 4. Conclusions

This work is a continuation of our research on the application of phosphate-bridged bisphthalonitrile monomer as a component of polymer matrix for composites produced by cost-effective injection methods. In our previous studies, we showed the positive impact of bis(3-(3,4-dicyanophenoxy)phenyl)phenyl phosphate on the technological properties of pthtalonitrile resins such as low melting point and wide processing window. In this work, we have studied the influence of the phosphate monomer content on the mechanical and physico-chemical properties of the thermosets as well as their thermal oxidative stability. Moreover, we have paid special attention to thermal oxidative aging experiments showing that TGA analysis is not enough to assess properly thermal oxidative stability of the material.

Based on the obtained results, it can be concluded that there is no difference in initial mechanical properties of the thermosets PN10–50. Herewith, the glass transition temperature increases with increase in PPhPN content (from 330 °C for PN-10 to 350 °C for PN-50) while TOS5% decreases in this raw (from 507 °C for PN-10 to 470 °C for PN-50). Also, the 10% of PPhPN content is critical for processability of the resin due to formation of a crystalline phase that makes it impossible to apply resin in injection methods.

Our long-term thermal oxidative aging experiments showed that during 200 h of aging at 300 °C, resins containing 10% and 15% of PPhPN exhibited minimal mass loss (approximately 1%) while retaining their initial mechanical properties. In contrast, resins with 30% and 50% content of PPhPN lost over 60% of their strength. The thermal degradation process in the studied resins as expected accelerated at 330 °C, yet PN10 and PN15 maintained flexural strength over 80 MPa after 48 h. Aging at 350 °C led to complete loss of mechanical strength across all the studied resins.

## Figures and Tables

**Table 1:** Component ratios of the studied phthalonitrile resins.

Sample	Mass, %		
	DPB	PPhPN	APN
PN50	15	50	35
PN30	35	30	35
PN15	50	15	35
PN10	55	10	35

**Table 2:** Characteristics of the phthalonitrile thermosets.

Thermoset	T_g_, °C	TOS_5%_, °C	Flexural Strength (σ_f_), MPa	Flexural Modulus (E_f_), GPa
PN10	330	507	141.8 ± 0.8	4.3 ± 0.1
PN15	327	507	138.6 ± 9.2	4.3 ± 0.1
PN30	352	498	140.2 ± 9.5	4.4 ± 0.1
PN50	350	470	140.0 ± 4.4	4.7 ± 0.1

**Table 3:** Glass transition temperatures of PN10–PN50 resins before and after 200 h of thermal oxidative aging.

Sample	Glass Transition Temperature (Tg), °C			
	Initial	Aging Temperature, °C		
		300	330	350
PN10	330	353	400	401 *
PN15	328	353	397	397 **
PN30	352	378	367 *	444 *
PN50	350	372	380 **	-

* The result after 100 h of aging; ** The result after 48 h of aging.

## Data Availability

Data are contained within the article and Supplementary Materials.

## Supplementary Materials

The following supporting information can be downloaded at: https://www.mdpi.com/article/10.3390/polym16162239/s1, Figure S1: DSC curve of the PN10 resin; Figure S2: DSC curves of the PN15, PN30 and PN50 resins; Figure S3: DMA curves of the PN10, PN15, PN30 and PN50; Figure S4: Mechanism of thermal degradation of organophosphates.

## References

[1] Derradji M., Jun W., Wenbin L., Wang J., Liu W. (2018). Phthalonitrile Resins and Composites: Properties and Applications.

[2] Sun B.G., Shi H.Q., Yang K.X., Lei Q., Li Y.Q., Fu Y.Q., Hu N., Guo Y., Zhou H., Fu S.Y. (2020). Effects of 3-aminophenylacetylene on mechanical properties at elevated temperatures of carbon fiber/phthalonitrile composites. Compos. Commun..

[3] Sun J., Han Y., Zhao Z., Wang G., Zhan S., Ding J., Liu X., Guo Y., Zhou H., Zhao T. (2021). Improved toughness of phthalonitrile composites through synergistic toughening methods. Compos. Commun..

[4] Yakovlev M.V., Kuchevskaia M.E., Terekhov V.E., Morozov O.S., Babkin A.V., Kepman A.V., Avdeev V.V., Bulgakov B.A. (2022). Easy processable tris-phthalonitrile based resins and carbon fabric reinforced composites fabricated by vacuum infusion. Mater. Today Commun..

[5] Lei W., Wang D., Li Y., Li K., Liu Q., Wang P., Feng W., Liu Q., Yang X. (2022). High temperature resistant polymer foam based on bi-functional benzoxazine-phthalonitrile resin. Polym. Degrad. Stab..

[6] Pu Y., Xie H., He X., Lv J., Zhu Z., Hong J., Zeng K., Hu J., Yang G. (2022). The curing reaction of phthalonitrile promoted by sulfhydryl groups with high curing activity. Polymer.

[7] Ji B., Pan Y., Lyu J., Liu J., Liao W., Yin C., Xing S., Wu N. (2023). Low defect and high mechanical properties POSS copolymerization phthalonitrile resin prepared by powder hot isostatic pressing. Mater. Lett..

[8] Ye J., Zhang S., Wu M., Liu X., Liu X. (2023). Thermal, mechanical and dielectric property enhancement of benzoxazine-containing phthalonitrile resin: The effect of functional oligomeric polyphenyl ether. Polymer.

[9] Li Q., Zhang S., Ye J., Liu X. (2022). Multiple catalytic polymerization of phthalonitrile resin bearing benzoxazine moiety: Greatly reduced curing temperature. Eur. Polym. J..

[10] Bulgakov B.A., Sulimov A.V., Babkin A.V., Afanasiev D.V., Solopchenko A.V., Afanaseva E.S., Kepman A.V., Avdeev V.V. (2017). Flame-retardant carbon fiber reinforced phthalonitrile composite for high-temperature applications obtained by resin transfer molding. Mendeleev Commun..

[11] Timoshkin I.A., Aleshkevich V.V., Afanas’eva E.S., Bulgakov B.A., Babkin A.V., Kepman A.V., Avdeev V.V. (2020). Heat-Resistant Carbon Fiber Reinforced Plastics Based on a Copolymer of Bisphthalonitriles and Bisbenzonitrile. Polym. Sci. Ser. C.

[12] Poliakova D., Morozov O., Lipatov Y., Babkin A., Kepman A., Avdeev V., Bulgakov B. (2022). Fast-Processable Non-Flammable Phthalonitrile-Modified Novolac/Carbon and Glass Fiber Composites. Polymers.

[13] Yang J., Wang D., Li M., Ji C., Wang B. (2023). Thermal response and pyrolysis behavior of carbon fiber/phthalonitrile composites under one-sided butane flame heating: Experimental and numerical analysis. Compos. Part A Appl. Sci. Manuf..

[14] Wang G., Han Y., Guo Y., Wang S., Sun J., Zhou H., Zhao T. (2019). Phthalonitrile-Terminated Silicon-Containing Oligomers: Synthesis, Polymerization, and Properties. Ind. Eng. Chem. Res..

[15] Pochiraju K.V., Tandon G.P. (2006). Modeling thermo-oxidative layer growth in high-temperature resins. J. Eng. Mater. Technol..

[16] Terekhov V.E., Morozov O.S., Afanaseva E.S., Bulgakov B.A., Babkin A.V., Kepman A.V., Avdeev V.V. (2020). Fluorinated phthalonitrile resins with improved thermal oxidative stability. Mendeleev Commun..

[17] Wang H., Zhang Z., Ji P., Yu X., Naito K., Zhang Q. (2019). Synthesis and properties of a novel high-temperature vinylpyridine-based phthalonitrile polymer. High Perform. Polym..

[18] Chaussoy N., Brandt D., Gérard J.F. (2023). Phthalonitrile functionalized resoles—Use of 2,3-dicyanohydroquinone as a versatile monomer for resins with very high thermal stability. Polym. Degrad. Stab..

[19] Zhang Z.-Q., Uth S., Sandman D.J., Foxman B.M. (2004). Structure, polymorphism and thermal properties of phenyliminoisoindolines. J. Phys. Org. Chem..

[20] Derradji M., Mehelli O., Khiari K., Abdous S., Soudjrari S., Zegaoui A., Ramdani N., Liu W., Al Hassan M. (2022). High performance green composite from vanillin-based benzoxazine containing phthalonitrile and silane surface modified basalt fibers. High Perform. Polym..

[21] Bulgakov B.A., Morozov O.S., Timoshkin I.A., Babkin A.V., Kepman A.V. (2021). Bisphthalonitrile-based Thermosets as Heat-resistant Matrices for Fiber Reinforced Plastics. Polym. Sci. Ser. C.

[22] Yin C., Zhang Y., Liao W., Liu J., Wu N., Xing S., Tang J. (2023). Improving mechanical properties of high-temperature resistant carbon fiber/phthalonitrile composites via surface modification: A comparative study on modification methods. Compos. Interfaces.

[23] Zhao D., Hu J., Wang D., Yang J., Zhang H., Wang B. (2023). Reinforcement of mica on phthalonitrile resin and composites: Curing, thermal, mechanical and dielectric properties. Compos. Sci. Technol..

[24] Yang X., Li Y., Lei W., Bai Z., Zhan Y., Li Y., Li K., Wang P., Feng W., Liu Q. (2023). Understanding the Thermal Degradation Mechanism of High-Temperature-Resistant Phthalonitrile Foam at Macroscopic and Molecular Levels. Polymers.

[25] Wu Z., Han J., Li N., Weng Z., Wang J., Jian X. (2017). Improving the curing process and thermal stability of phthalonitrile resin via novel mixed curing agents. Polym. Int..

[26] Hu Y., Weng Z., Qi Y., Wang J., Zhang S., Liu C., Zong L., Jian X. (2018). Self-curing triphenol A-based phthalonitrile resin precursor acts as a flexibilizer and curing agent for phthalonitrile resin. RSC Adv..

[27] Ye J., Li Q., Zhang S., Liu X. (2022). Melamine modified phthalonitrile resins: Synthesis, polymerization and properties. Polymer.

[28] Kolesnikov T.I., Orlova A.M., Tsegelskaya A.Y., Cherkaev G.V., Kechekyan A.S., Buzin A.I., Dmitryakov P.V., Belousov S.I., Abramov I.G., Serushkina O.V. (2021). Dual-curing propargyl-phthalonitrile imide-based thermoset: Synthesis, characterization and curing behavior. Eur. Polym. J..

[29] Babkin A.V., Zodbinov E.B., Bulgakov B.A., Kepman A.V., Avdeev V.V. (2016). Thermally stable phthalonitrile matrixes containing siloxane fragments. Polym. Sci. Ser. B.

[30] Laskoski M., Dominguez D.D., Keller T.M. (2007). Synthesis and properties of aromatic ether phosphine oxide containing oligomeric phthalonitrile resins with improved oxidative stability. Polymer.

[31] Monzel W.J., Lu G.-Q.Q., Pruyn T.L., Houser C.L., Yee G.T. (2019). Thermal and oxidative behavior of a tetraphenylsilane-containing phthalonitrile polymer. High Perform. Polym..

[32] Bulgakov B.A., Babkin A.V., Dzhevakov P.B., Bogolyubov A.A., Sulimov A.V., Kepman A.V., Kolyagin Y.G., Guseva D.V., Rudyak V.Y., Chertovich A.V. (2016). Low-melting phthalonitrile thermosetting monomers with siloxane- and phosphate bridges. Eur. Polym. J..

[33] Wu M., Gu Y., Hao D., Chen X., Yu X., Zhang Q. (2022). Fluorinated Phthalonitrile Resin with Excellent Thermal Stability and Low Dielectric Constant for High-Frequency Electronic Packaging. Macromol. Mater. Eng..

[34] Morgan A.B., Putthanarat S. (2011). Use of inorganic materials to enhance thermal stability and flammability behavior of a polyimide. Polym. Degrad. Stab..

[35] Li Z., Guo Y., Wang G., Xu S., Han Y., Liu X., Luo Z., Ye L., Zhou H., Zhao T. (2018). Preparation and characterization of a self-catalyzed fluorinated novolac-phthalonitrile resin. Polym. Adv. Technol..

[36] Dai X., Li P., Sui Y., Zhang C. (2021). Thermal and flame-retardant properties of intrinsic flame-retardant epoxy resin containing biphenyl structures and phosphorus. Eur. Polym. J..

[37] Özer M.S., Gaan S. (2022). Recent developments in phosphorus based flame retardant coatings for textiles: Synthesis, applications and performance. Prog. Org. Coat..

[38] Wang W., Liao C., Liu L., Cai W., Yuan Y., Hou Y., Guo W., Zhou X., Qiu S., Song L. (2019). Comparable investigation of tervalent and pentavalent phosphorus based flame retardants on improving the safety and capacity of lithium-ion batteries. J. Power Sources.

[39] Xu W., Wang G., Zheng X. (2015). Research on highly flame-retardant rigid PU foams by combination of nanostructured additives and phosphorus flame retardants. Polym. Degrad. Stab..

[40] Nazir R., Gooneie A., Lehner S., Jovic M., Rupper P., Ott N., Hufenus R., Gaan S. (2021). Alkyl sulfone bridged phosphorus flame-retardants for polypropylene. Mater. Des..

[41] Zhang W., Zhou M., Kan Y., Chen J., Hu Y., Xing W. (2023). Synthesis and flame retardant efficiency study of two phosphorus-nitrogen type flame retardants containing triazole units. Polym. Degrad. Stab..

[42] Terekhov V.E., Aleshkevich V.V., Afanaseva E.S., Nechausov S.S., Babkin A.V., Bulgakov B.A., Kepman A.V., Avdeev V.V. (2019). Bis(4-cyanophenyl) phenyl phosphate as viscosity reducing comonomer for phthalonitrile resins. React. Funct. Polym..

[43] Lobanova M.S., Aleshkevich V.V., Yablokova M.Y., Morozov O.S., Babkin A.V., Kepman A.V., Avdeev V.V., Bulgakov B.A. (2023). Kinetics of the oxidative aging of phthalonitrile resins and their effects on the mechanical properties of thermosets. Thermochim. Acta.

[44] Lobanova M., Aleshkevich V., Babkin A., Kepman A., Avdeev V., Morozov O., Bulgakov B. (2023). Effect of post-curing temperature on the retention of mechanical strength of phthalonitrile thermosets and composites after a long-term thermal oxidative aging. Polym. Compos..

[45] Keller T.M., Dominguez D.D. (2005). High temperature resorcinol-based phthalonitrile polymer. Polymer.

[46] Cao G.P., Chen W.J., Liu X.B. (2008). Synthesis and thermal properties of the thermosetting resin based on cyano functionalized benzoxazine. Polym. Degrad. Stab..

[47] (2017). Standard Test Methods for Flexural Properties of Unreinforced and Reinforced Plastics and Electrical Insulating Materials.

[48] Shankwalkar S.G., Cruz C. (1994). Thermal Degradation and Weight Loss Characteristics of Commercial Phosphate Esters. Ind. Eng. Chem. Res..

[49] Liu C., Yao Q. (2018). Mechanism of thermal degradation of aryl bisphosphates and the formation of polyphosphates. J. Anal. Appl. Pyrolysis.

[50] Aleshkevich V., Morozov O., Babkin A., Kepman A., Bulgakov B. (2024). High-performance C/C composites derived from phthalonitrile matrix CFRP via a few cycles of vacuum-assisted impregnation-carbonization. Compos. Part A.

[51] Buch X., Shanahan M.E.R. (2000). Thermal and thermo-oxidative ageing of an epoxy adhesive. Polym. Degrad. Stab..

[52] Liu Y.L., Hsiue G.H., Lan C.W., Chiu Y.S. (1997). Phosphorus-containing epoxy for flame retardance: IV. Kinetics and mechanism of thermal degradation. Polym. Degrad. Stab..

