# Supplementary material for: Effect of Phosphate-Bridged Monomer on Thermal Oxidative Behavior of Phthalonitrile Thermosets
Source: Polymers (Basel). 2024 Aug 7;16(16):2239. doi: 10.3390/polym16162239 (PMC11359510; doi:10.3390/polym16162239)
Supplement: Supplementary file 1 [file polymers-16-02239-s001.zip › polymers-3113715-supplementary.pdf]

polymers

Supplementary Information for

Effect of Phosphate-Bridged Monomer on Thermal Oxidative Behavior of Phthalonitrile Thermosets

Marina Sergeevna Lobanova *, Alexandr Vladimirovich Babkin, Alexey Valeryevich Kepman, Victor Vasil'evich Avdeev, Oleg Sergeevich Morozov and Boris Anatol'evich Bulgakov

Department of Chemistry, M. V. Lomonosov Moscow State University, 119991 Moscow, Russia
* Correspondence: lobanovams8@gmail.com

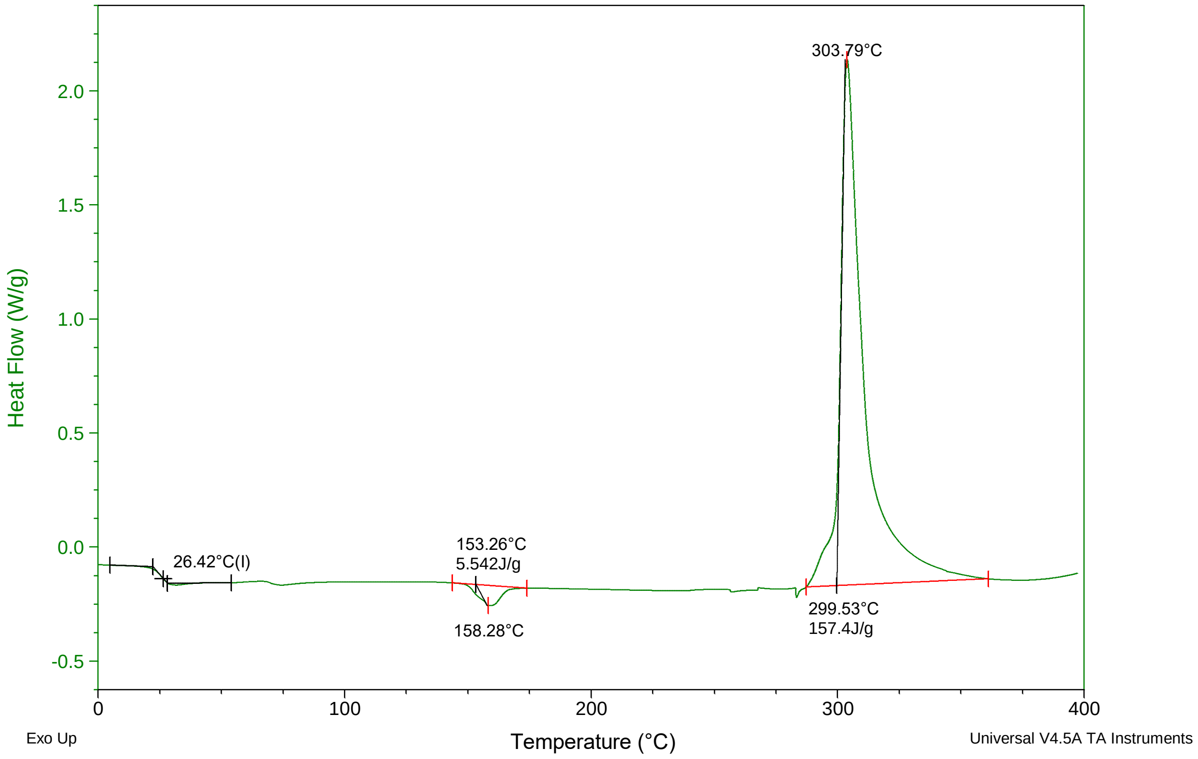

Figure S1. DSC curve of the PN10 resin.

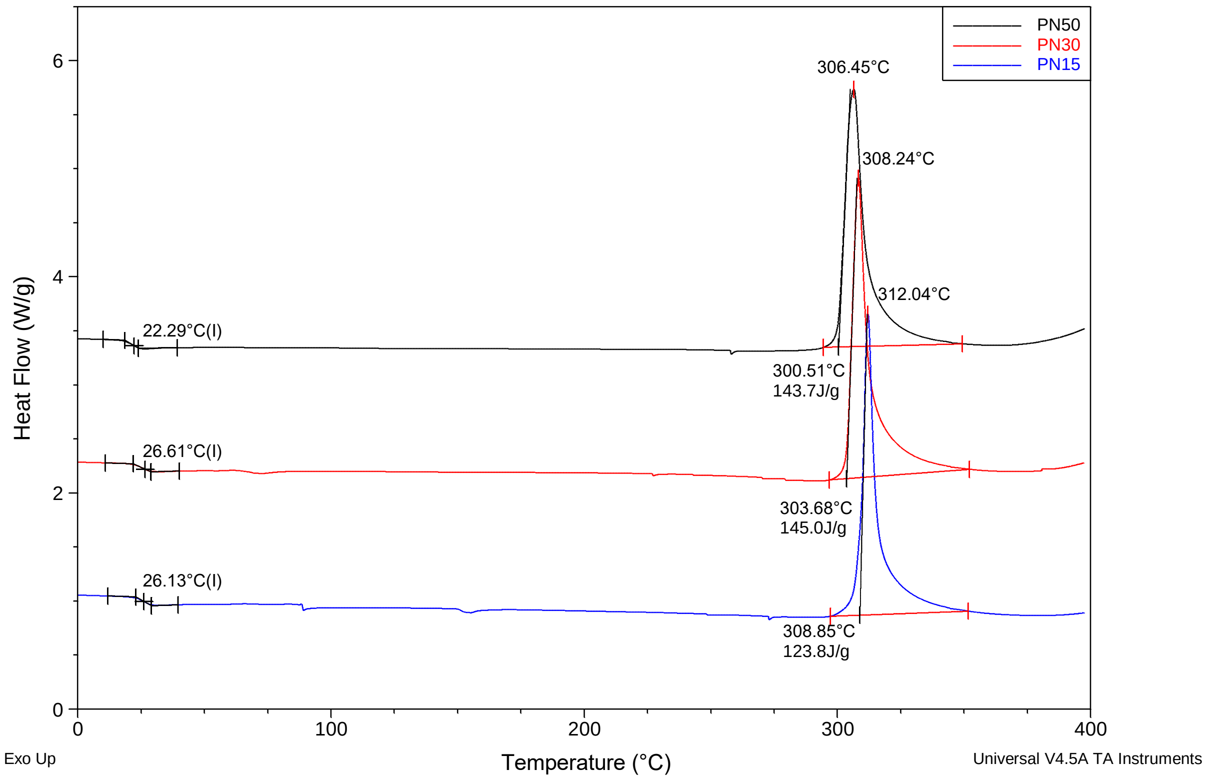

Figure S2. DSC curves of the PN15, PN30 and PN50 resins.

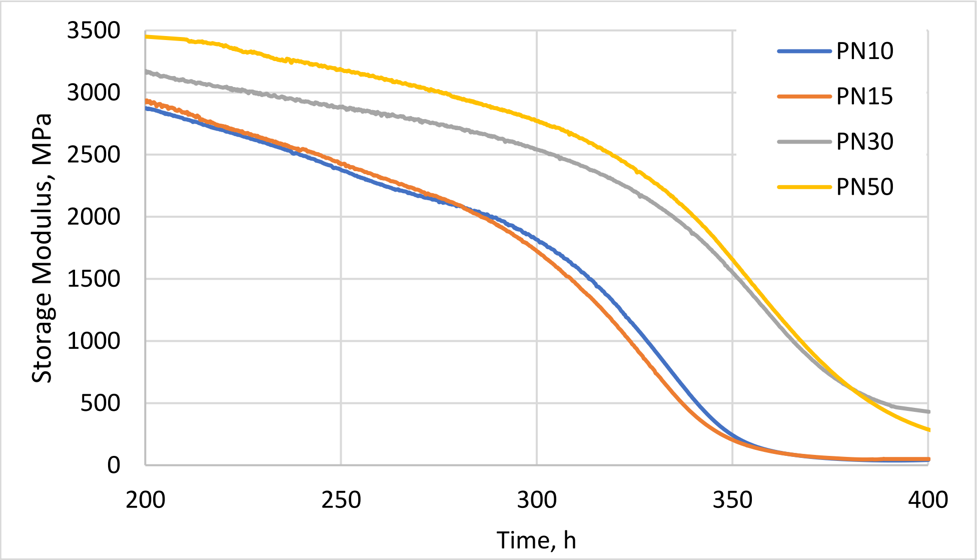

Figure S3. DMA curves of the PN10, PN15, PN30 and PN50.

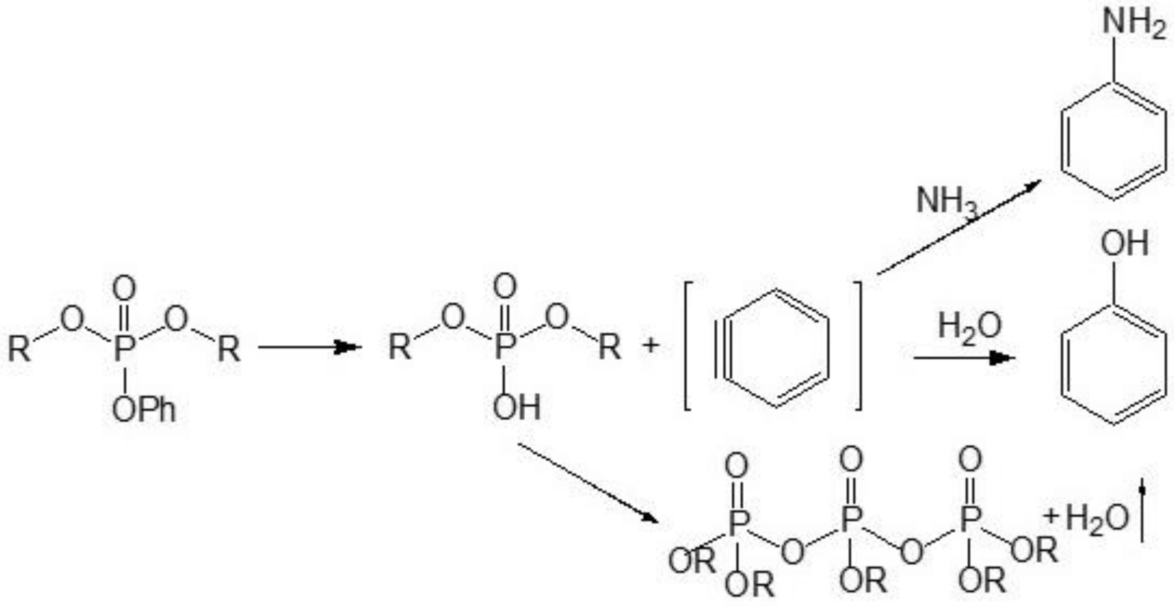

Figure S4. Mechanism of thermal degradation of organophosphates.
